# Sequence and Structural Analysis of 3' Untranslated Region of Hepatitis C Virus, Genotype 3a, From Pakistani Isolates

**DOI:** 10.5812/hepatmon.8390

**Published:** 2013-05-09

**Authors:** Sadia Anjum, Sidra Ali, Tahir Ahmad, Muhammad Sohail Afzal, Yasir Waheed, Talha Shafi, Muhammad Ashraf, Saadia Andleeb

**Affiliations:** 1 Atta-ur Rahman School of Applied Biosciences, National University of Sciences and Technology, Islamabad, Pakistan

**Keywords:** Hepacivirus, Phylogeny, Untranslated Regions, Multilocus Sequence Typing

## Abstract

**Background:**

Hepatitis C virus (HCV) is the cause of high morbidity and mortality worldwide, inflicting around one million people in Pakistan alone. The HCV genomic RNA harbors conserved structural elements that are indispensable for its replication. The 3’ untranslated region (UTR) contains several of these elements essentially involved in regulating the major steps of the viral life cycle.

**Objectives:**

Differences in regulatory elements of HCV may contribute towards differential infectivity of local isolates. The present study explicates sequence analysis and secondary structure prediction of HCV 3'UTR region of subtype 3a from Pakistan to characterize this particular region.

**Patients and Methods:**

HCV 3'UTR region was amplified, cloned and sequenced from five different patients. Sequence and structural analysis was performed and phylogenetic analysis was carried out using the 3'UTR sequence reported in NCBI nucleotide data base (http://www.ncbi.nlm.nih.gov/nuccore) by other studies.

**Results:**

Sequence analysis of the amplified fragment from five patients indicated that the 3'UTR is composed of 214-235 nts. Its sequence contains a type-specific variable region followed by a poly U/UC region and a highly conserved X-tail of 98 nts. The variable region reported here has 26 nts and one stem loop at the secondary structure that differentiate it from HCV genotype 1a ( GT1a) 3'UTR which contains additional 14 nts and two stem loops. The poly U/UC region varied in length (100-79 nts) and nucleotide sequence within the Pakistani isolates, and among different genotypes. Some substitutions found in the X-tail do not affect secondary structure of this element suggesting that this region might play an important role in replication, stabilization and packaging of HCV genome. Additionally, U residues are not present at the end of the X-tail in Pakistani 3a isolates as otherwise reported for the variants of genotype 1b.

**Conclusions:**

Sequence and structural diversity of the 3'UTR variable region and Poly U/UC region found in the local isolates indicate specificity in the regulating elements of 3'UTR that might be associated with differential replication efficacy of the HCV Pakistani isolates. The study necessitates functional characterization of these regulating elements to elucidate variable viral efficiency and pathogenicity associated with inter-geographical isolates.

## 1. Background

The molecular cloning of Hepatitis C Virus (HCV) was reported in 1989 (1). Since then sequence analysis of HCV isolates has unraveled genetic diversities among the multiple genotypes. Hepatitis C virus genome is about ~9.4 kb in length encoding at least 10 structural and non-structural proteins flanked by two cis-acting RNA elements known as the 5' and 3' untranslated regions. HCV genomic RNA serves as mRNA for production of a polyprotein that is co- and post-translationally modified by cellular and viral proteases. HCV translation starts from a highly conserved 5'-untranslated region (5'UTR), which functions as an Internal Ribosomal Entry Site (2, 3) that function in a cap-independent mode for initiation of translation. The 3'-untranslated region located at the end of the viral genome (4) is a tripartite structure and is predominantly involved in HCV replication (5, 6). This region is essentially required for viral RNA replication, either in chimpanzees inoculated with synthetic genome-length RNA or in HCV replicon in a cell culture system (5-7). The tripartite structure of the 3'UTR is composed of a variable region of 26-70nt length, a polyU/UC tract of variable length and sequence, and the X-tail of 98-100 nts which is conserved among various genotypes (8, 9). The 3'UTR contain signals that are indispensable for and regulate viral RNA replication besides their role in HCV translation. The sequence and secondary structure of this region is highly conserved among different genotypes and subtypes (10). The interaction of 5'UTR, 3'UTR and NS5B is mandatory for viral replication (11) particularly in the initiation of minus-strand RNA (7). 3'UTR predicted stem-loop structure located at the 3’ terminus (nts 5 to 20 from the 3’ end) of the negative-strand RNA, is reported to interact with NS3. This interaction is thought to lead RNA-protein complexes to the cytoplasmic membranes where viral replication complexes are formed (12). Binding of NS5A with 3'UTR is expected to mediate genome circularization and switch on the viral translation (13). Likewise binding of PTB (TATA binding protein) to both 5′ and 3’ cis-elements within the UTRs has been proposed to block the initiation of replication from the 3’UTR by exerting a positive influence on the IRES while preventing NS5B from binding to the 3’UTR needed for the assembly of the replication initiation complex (14). In addition, IGF2BP1 binds to the HCV 5'UTR and or HCV 3'UTR, recruits eIF3 and enhances HCV IRES-mediated translation (15). HCV RNA stability is also affected by 3'UTR because it is known to bind with Human liver protein La, an auto antigen which protects RNA from degradation (16). RNA-RNA interactions between the 5'-3'ends of HCV genome play an important role; molecular interaction of domain IIId of IRES, and SLII and SLIII of the 3'UTR as well as the CRE-region found at 3' end of NS5b modulate translational efficiency and also the switch between RNA translation and replication (17). 3'X-tail within the 3’UTR is confidently employed in HCV detection and quantification by sequencing the X-tail from a broad and complete panel of HCV genotypes. A distinct feature of the 3'UTR is the absence of its cellular homologue and this makes it an important therapeutic target (4). Mutations in 3'UTR can affect protein binding which may be associated with disease in humans (17). Approximately more than 10 million people are suffering from HCV infections in Pakistan, more than 50% of them are with genotype (GT) 3a (18, 19). The major reason for high infection is due to poor medical practices (20).

## 2. Objectives 

The aim of the current study was to identify sequence and structural variability in the 3’UTR region of GT3a isolates from Pakistan, to develop a better understanding of HCV life cycle and replication mechanism. Studying this particular region of the virus needs to be extended as this region is expected to play an essential role in viral replication and very limited data is available regarding this region of the virus. This study will add important information and will help predict new therapeutic or drug targets specifically against replication.

## 3. Patients and Methods

HCV, genotype 3a positive patients, confirmed by ASAB diagnostic, were enrolled for this study under approval of the Internal Review Board (IRB) of ASAB, NUST, Pakistan and a patient consent form was duly signed for each patient. All these patients were strictly non IV drug users and the potential risk factor was either minor surgery or dental treatment. The details of these patients is given in Table 1.

**Table 1.** Information on Participating Patients

Patient, Number	ASAB Diagnostic,Identification Number	Sex	Age, y^a^	ViralRNALoad, Copies/mL
**1**	5347	Male	62	564776163
**2**	2712	Female	38	78792381
**3**	3949	Male	39	724643779
**4**	3897	Male	40	979291553
**5**	9600	Female	50	995891553

^a^Abbreviations: y, year

### 3.1. Extraction of Nucleic Acid 

Patient’s blood samples (1300 µl) were taken in EDTA vacutainer tubes. The samples were centrifuged at 12000 g for 2 min to isolate serum. Viral RNA was extracted from serum by Qiagen RNA extraction kit according to the manufacturer protocols (Qiagen, Germany, Hamburg). RNA was stored at -20°C.

### 3.2. Complementary DNA (cDNA) Synthesis and PCR Amplification

For PCR amplification of the HCV 3'UTR region, primers were designed by retrieving sequences of the specific viral genes of NZL1, S52 and HCV-K3A isolates from NCBI Nucleotide database. The sequence of the sense primer used was “TGAGCTGGTAGGATAACACTCC” and the antisense primer was “ACATGATCTGCAGAGAGGCCAGTATC” and the PCR amplified product size was expected to be 235 base pairs. Extracted RNA was used as a template for the cDNA synthesis. Reverse transcription was carried out in 20µl volume of the reaction mix, containing 13µl of RNA, 0.2mM dNTPs, 1X MMulv Buffer, 20 units of molony murine leukemia virus reverse transcriptase enzyme (Fermentas, Canada) and 2 pmol specific antisense primer. The reaction was carried out at 42°C for 55 min followed by 70°C for 10 min. PCR reactions were performed by using 5 µl of cDNA as template, 2 pmol each of sense and antisense primer, 0.4 mM of DNTPs, 1X of high fidelity long mix buffer (Roche, France), 2 units of high fidelity long mix enzyme and nuclease free water to make up the volume. The cycling conditions were optimized; Initial denaturation step at 94˚C for 3 minutes, 30 cycles of 45 seconds denaturation at 94˚C, 45 seconds for annealing at 58˚C, 3 minute for extension at 68°C, and a final extension at 68˚C for 10 minutes. PCR products were purified by Qiagen gel extraction kit (promega, China).

### 3.3. Cloning and Sequencing

Purified PCR products were cloned in PCRII TOPO cloning vector (Invitrogen, Singapore) as instructed by the manufacturer and ligation products were transformed in Top 10 bacterial cells. The principal of blue white selection was used for initial screening and positive clones were then confirmed by restriction analysis. Clones were subjected to sequencing by using Beckman coulter CEQ 8000 (USA). The sequencing reaction contained 200 ng template DNA, 6 ul of water, 10 pmol T7 promoter specific forward and reverse primer and 8 µL of dye terminator cycle sequencing (DTCS) mix. The thermo cycler conditions for sequencing reaction were 96°C for 20 s, 50°C for 20 s, 60°C for 4 min for 30 cycles followed by final hold at 4°C. Five ul of stop solution containing 2 ul of 3 M sodium acetate, 2 ul of 100 mM disodium EDTA and 1 ul of 20 mg/ml of glycogen was added to each tube. The sequencing reaction containing stop solution was then washed with 100% followed by 70% ethanol and was vacuum dried. The pellet was resuspended in 40 ul of sample loading solution and was subjected to sequencing reaction.

### 3.4. In Silico Analysis of Sequenced Product

**Figure 1.**
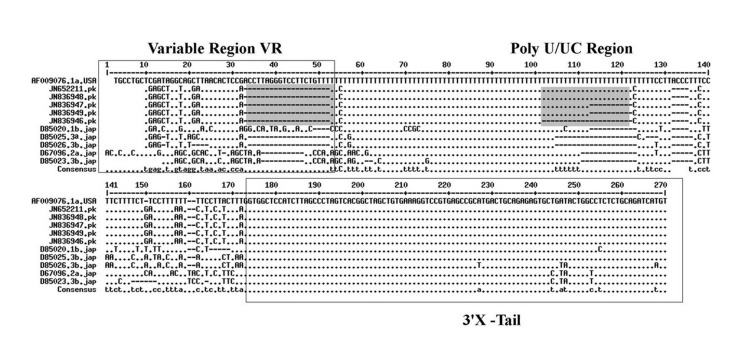
Multiple Sequence Alignment of HCV 3’UTR With Reference Strain H77 1a (AF009076) and Others Using MultAlin Interface Page (multalin.toulouse.inra.fr) Nucleotide deletions in Pakistani isolates reported in this study are shown in grey boxes and substitutions are shown by letters (nucleotides) while the dot represents the conserved nucleotides are for missing nucleotides. Major changes in 3’UTR of Pakistani isolates are seen in variable region (grey rectangle represents the missing loop in VR) and poly U/UC region, which is of variable length in each isolate (grey rectangle).

Three sequences from each clone were obtained and aligned in the CLC workbench software (www.clcbio.com) to compile consensus sequences and the consensus sequences were submitted to NCBI. Sequences of HCV 3'UTR reported from all over the world were retrieved from NCBI nucleotide databank. Multiple sequence alignment of 3'UTR sequences from the present study and others reported earlier from different parts of the world (Japan, USA) was done by using the MultAlin Interface page (Figure 1). Secondary structure of HCV 3'UTR was predicted using the M-fold web server (http://mfold.rna.albany.edu) based on minimal free energy algorithm (21). Phylogenetic tree was constructed using PhyML (Phylogenetic Maximum Likelihood) using http://www.atgc-montpellier.fr online web server (22).

## 4. Results

### 4.1. Sequence Analysis and Secondary Structure Prediction

**Figure 2.**
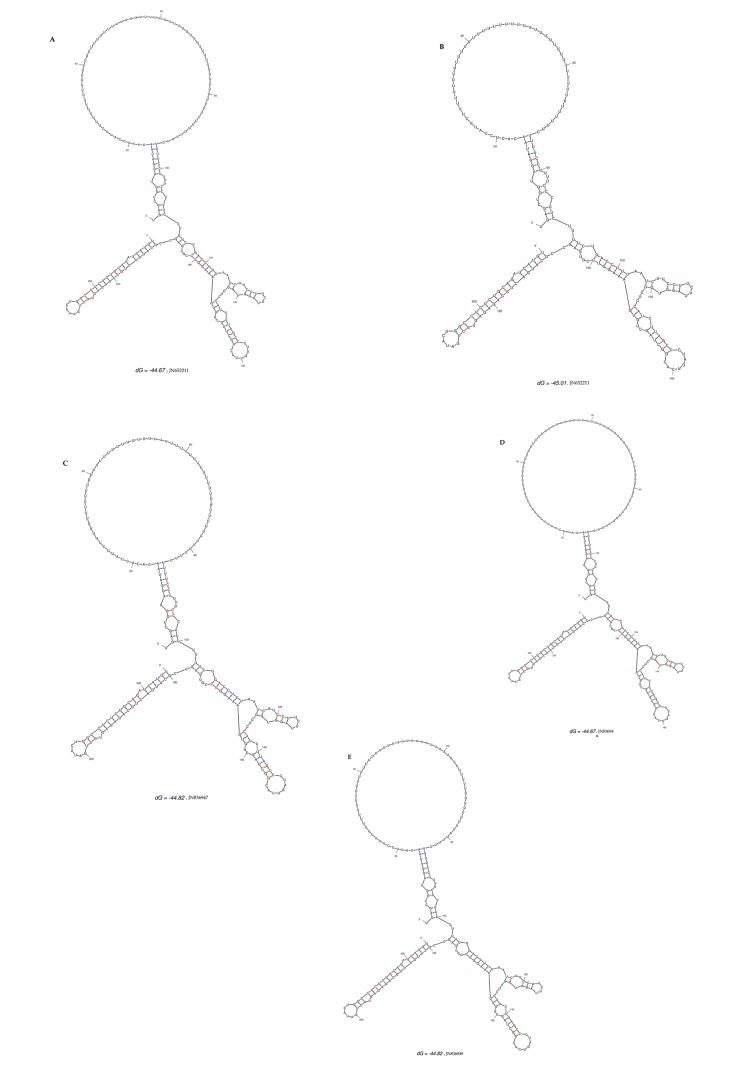
Predicted Secondary Structures of the 3'UTR for the Five Different HCV 3a Samples by Using the M fold Web Server (mfold.rna.albany.edu) Minimum free energy varied from Dg = -44.67 cal/mol (A, E), -44.82 cal/mol (C, D) to -45.01 cal/mol (B). The accession number of each clone is shown at the bottom.

HCV 3'UTR region from five different patients was amplified and cloned. Three clones were obtained from each patient. These clones were sequenced and their consensus sequences were submitted to the NCBI data base under accession numbers JN652211, JN836946, JN836947, JN836948 and JN836949. For sequence analysis, sequences of HCV 3'UTR were retrieved from NCBI Databank. Then, multiple sequence alignment of 3'UTR was performed and the data revealed that 3'-untranslated region was found to be 214-235 nts in length from Pakistani isolates (Figure 1). The results showed 98% similarity among intra-genotypic isolates and 87% homology among inter-genotypic isolates reported from around the globe. Although conservation is maintained in this part of the genome, different genotypes do exhibit some nucleotide variations. For further analysis, secondary structure of HCV 3'UTR was predicted using M fold web server and the results confirmed the presence of three distinct regions i.e., a variable region (VR), a poly U/UC tract of variable length and sequence and a highly conserved 3'X-tail (Figures 2 A, B, C, D, E).

**Figure 3.**
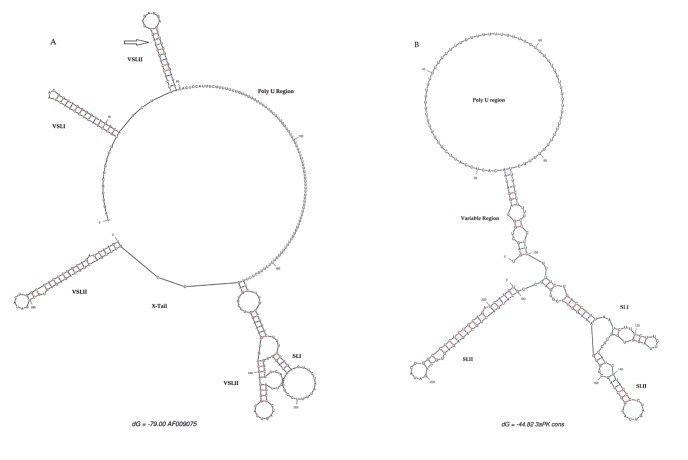
Secondary Structure of the 3’UTR Region A) shows secondary structure of subtype1a (AF009075) and B) shows secondary structure of subtype 3a derived from the consensus sequence of the Pakistani isolate. The arrow indicates the missing VSLII from Pakistani isolates.

### 4.2. Phylogenetic Analysis 

**Figure 4.**
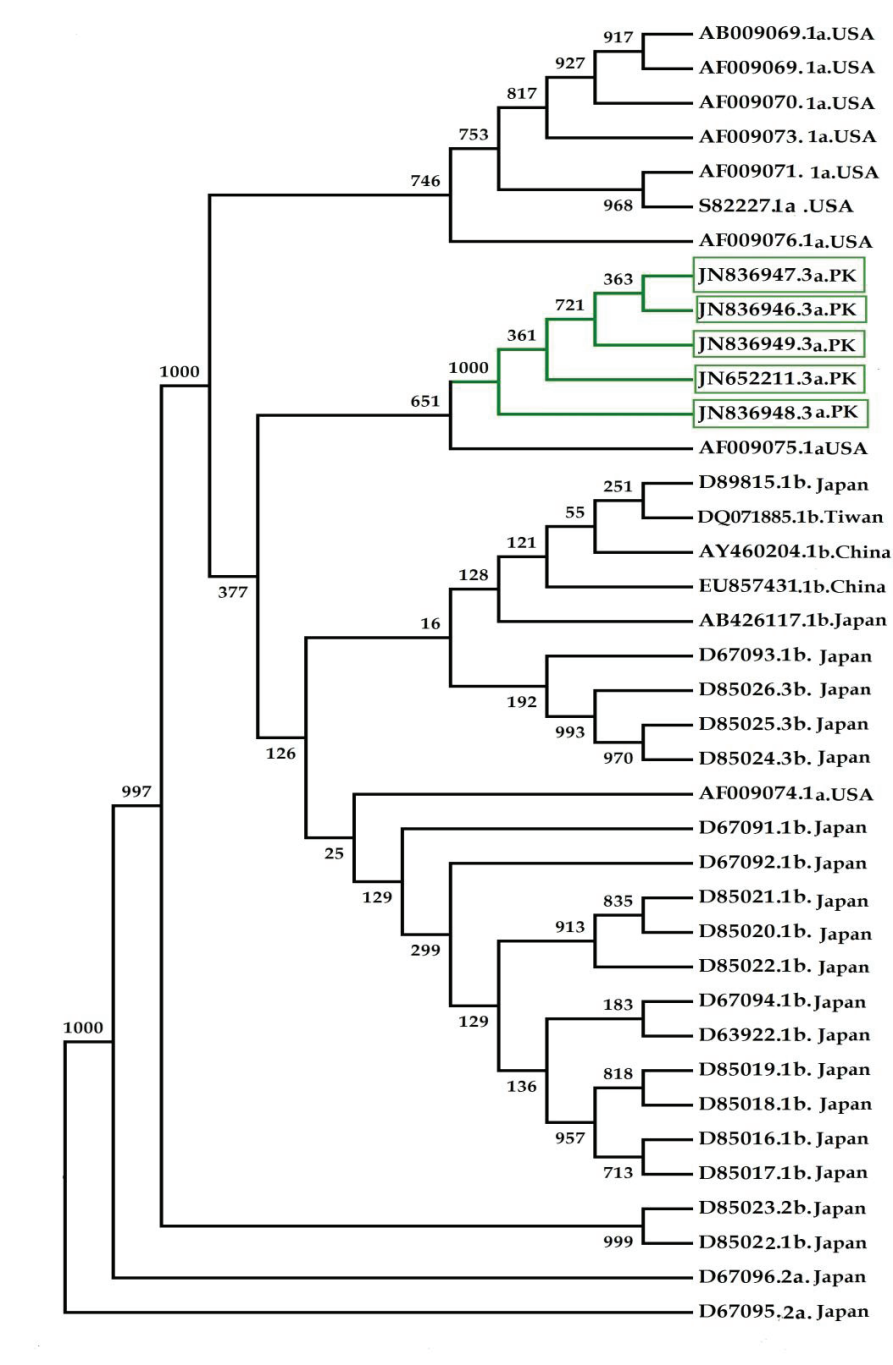
HCV 3'UTR Sequence Based Phylogenetic Tree Constructed UsingPhyML (Phylogenetic Maximum Likelihood) Using http://www.atgc-montpellier.fr Online Web Server 1000 replicates were used for this purpose; Bootstrap values up to 1000 are indicated on each branch; 3'UTR sequences from the present study are shown in green.

Phylogenetic analysis of the HCV 3’UTR region was performed for Pakistani isolates with other sequences reported from the rest of the world (Figure 4). Results indicated an independent origin of HCV all over the world. All Pakistani isolates included in the phylogenetic analyses were grouped together showing their high resemblance.

## 5. Discussion

3’ untranslated region of HCV genome is slightly variable among different genotypes. HCV replicates with different recognition signals at the 3’-end and this may contribute to differences in virulence of different genotypes and their replicative efficiency in cell culture system (23). Results of cloning and sequencing of HCV 3’ untranslated region from five infected patients showed 98% similarity among intra-genotypic isolates and 87% homology among inter-genotypic isolates reported from other countries, showing that, although conservation is maintained in this part of the genome, different genotypes do exhibit some nucleotide variations. The nucleotide sequence of this region confirms the presence of three distinct regions i.e., a variable region (VR), a poly U/UC tract (of variable length and sequence in five different samples) and a highly conserved 3'X-tail as reported previously (8 , 24). The length of variable region is 26 nucleotides and its sequence was conserved in all 5 clones, length of Poly U/UC region was different, two clones JN652211 and JN836948 have 111 nucleotides in this region, while JN836947 and JN836949 have 101 nucleotides, a single clone JN836946 had 90 nucleotides (Figure 1). The X-tail was the most conserved part, 98 nucleotides in length. A domain ACACUCC at position 16 and a UUC motif representing the last three nucleotides of variable region as previously described for the HCV-3a strain S52 (25) were found conserved in all of our clones. Additionally no extra U residues were found at the end of the X-tail in our 3a isolates, as otherwise reported for the variants of HCV 1b genotype. To further analyze, multiple sequence alignment of clones (accession numbers JN836946, JN836947, JN836949, JN652211, JN836948) was carried out with the HCV genotype 1a 1b, 2a, 2b, 3a, 3b isolates (Figure. 1). Results showed that the variable region is significantly different in length and nucleotide sequence among different genotypes whereas it is conserved among the same genotype. Alignment results showed that reference strain HCV H77-1a has a 10 nucleotide sequence at the end of the variable region (VR); this region is found to be missing in all of our clones and the one reported from Japan with subtype 3a (accession number D85025). The length and sequence of Poly U/UC region is different among our clones, varying from 100 nts (JN836948, JN652211), 91nts (JN836949 JN836947), to 79 nts (JN836946). This finding is in consistency with the Poly U/UC tract of variable length in earlier reported sequences of 1a and 1b (25, 26 ) and a homouridine sequence with a minimum of 26 residues are essential for effective replication in cell culture (5). Interestingly the length of poly U/UC tract is associated with the replication efficiency of the virus in chimpanzees as HCV with a longer poly U/UC region had a replicative advantage over the one with a shorter poly U/UC region (27). While the last region, 3'X tail, was found to be 98 nts and was conserved in all of the different genotypes giving possible evidence for its involvement in replication, stability (6, 28) and translation of HCV polyprotein (29). Secondary structure for this RNA molecule was predicted using the Mfold software, which is a dynamic programming software based on minimizing free energies (MFE) and provides the thermodynamically favored structure. The predicted structure contained one stem loop in the variable region and three stem loops in the X-tail with a minimum free energy of -52 kcal/mol (Figure 2, 3). Although two stem loops are reported for the variable region in the 3'UTR ( 4 , 5) a missing stem loop in the variable region in our isolates (Figure 3) might have some subtype specific functional importance that needs to be investigated. The varying length of Poly U/UC does not affect its overall secondary structure (Figure 2, 3). Despite the minor substitutions, the overall secondary structure of 3'X-tail is conserved as the stem loops I, II and III in this part have been reported previously to be essential for in vivo infectivity (27, 30, 31). To analyze the phylogenetic relationship between our Pakistani HCV isolates and other strains reported from different parts of the world, 3'UTR sequences were retrieved from the HCV data base. There are a few 3'UTR sequences in the data base and the rest of them were taken from the full genome. There are a few reliable full genome sequences available. Only those sequences that showed reliable results are included in the phylogenetic analysis. The phylogenetic trees were constructed using the maximum likelihood method (22) . The tree constructed clustered Pakistani isolates together (Figure 4) showing their close relationship with 1a USA and Japanese subtype 1b. Recent reports from Pakistan also suggest a phylogenetic link between Pakistani HCV isolates and Japanese HCV isolates for genes like the NS4a region (32) core region (33) and E1, E2 region (unpublished data from our lab). There are a few reports on the structural and functional motifs of HCV 3'UTR. However, from protein binding and deletion mutant studies, it is now believed that this cis-acting element is indispensable for viral replication. Our study suggests that there is a vital need for extensive characterization of this region from all genotypes. It would be of great interest to evaluate the functional role of genetic diversities existing in variable and poly U/UC region of 3'UTR. We propose that these variations might be responsible for heterogenic viral efficiency and pathogenicity associated with inter-geographical and inter-genotypic isolates. To the best of our knowledge, the current study is the first report on 3' untranslated region from HCV genotype 3a, from Pakistan. We have observed a missing stem loop in the variable region of our isolates that might be associated with the relatively high rate of HCV infection in Pakistan. In addition polyU/UC region was found to be variable in length and sequence both inter-genotypically and intra-genotypically. These variations may be associated with replication efficiency of individual HCV isolates and possibly play an important role in variable outcomes of the disease seen in various patients infected with the same genotype. Since the X-tail reported here is highly conserved among all genotypes, suggesting its imperative role in replication and proving it to be a potential target of RNA interacting drugs. This study necessitates functional characterization of these HCV regulating elements to elucidate variable viral efficiency and pathogenecity associated with inter-geographical isolates.
